# Identification, sexual dimorphism, and allometric effects of three psyllid species of the genus *Psyllopsis* by geometric morphometric analysis (Hemiptera, Liviidae)

**DOI:** 10.3897/zookeys.737.11560

**Published:** 2018-02-12

**Authors:** Roghayeh Shamsi Gushki, Mohammadreza Lashkari, Saeid Mirzaei

**Affiliations:** 1 Department of Biodiversity, Institute of Science and High Technology and Environmental Sciences, Graduate University of Advanced Technology, Kerman, Iran; 2 Department of Biotechnology, Institute of Science and High Technology and Environmental Sciences, Graduate University of Advanced Technology, Kerman, Postal Code: 7631133131, P.O.Box : 76315-117, Iran

**Keywords:** Diaphorinini, diversity, Euphyllurinae, jumping plant lice, Liviidae

## Abstract

Jumping plant lice (Hemiptera: Psylloidea) are considered important vectors of plant diseases and also economically important pests in agriculture and forest ecosystems. Three psyllid species *Psyllopsis repens* Loginova, 1963, *Psyllopsis securicola* Loginova, 1963, and *Psyllopsis machinosus* Loginova, 1963 associated with the ash tree *Fraxinus* are morphologically very similar. So far, their distinction has been possible only by comparing their male and female genitalia. In this research, forewing shape and size characteristics, sexual dimorphism and their allometric effects, using geometric morphometric analysis, were examined for identification purposes. The results showed significant differences in wing shape and size between the species studied. Based on the results, two species P. m*achinosus* and *P. securicola* can be differentiated with the vein M1+2, as in *P. securicola* the vein M1+2 is located between Rs and M3+4 veins, but the vein M1+2 is closer to the vein M3+4 in *P. machinosus*; also, *P. repens* can be differentiated from the two species *P. machinosus* and *P. securicola* by vein M. Hence, the veins M1+2, M3+4, Rs and M were the most important wing characters for discrimination of the three species, especially in the field. The analysis also showed significant differences in wing shape and size between male and female of the three species, and the allometric analysis showed that significant shape differences still remain in constant size in P. m*achinosus* and *P. repens*. Geometric changes in the forewings of both sexes for the three species are illustrated.

## Introduction

Psyllids (Hemiptera: Psylloidea) are considered as important vectors of plant diseases and economically important pests in agricultural and forest ecosystems (Burckhardt and Ouvrard 2012). Certain psyllid species have also been proposed as potential biocontrol agents for weed control (Burckhardt and Ouvrard 2012). So far, 3850 species of psyllids have been described (Li 2011); of these, 95 species belonging to 26 genera and 5 families have been recorded in Iran.

*Psyllopsis* Low, 1879 is a small Palaearctic genus comprised of 11 species in the world, which are introduced with various species of ash (*Fraxinus* spp., Oleaceae). *Psyllopsis* genus is currently classified in the family Liviidae, sub-family Euphyllurinae, and tribe Diaphorinini (Burckhardt and Ouvrard 2012). Psyllid adults and nymphs suck sap from plants and gradually cause marginal leaf rolling and gall forming. These areas gradually become brown and severely damage the tree.

Among the genus *Psyllopsis*, several species are distributed in Central Asia, such as *Psyllopsis repens* Loginova, 1963, which has been known from Afghanistan, Armenia, Azerbaijan, Caucasus, Iran, Serbia, Turkey (Ouvrard 2016) and Europe (from Belgrade in Serbia) (Malenovský and Jerinić-Prodanović 2011). Another species *Psyllopsis securicola* Loginova, 1963 is also distributed in Iran, Afghanistan, Armenia, Azerbaijan, Caucasus, Georgia, Iraq, Tadzhikistan, Turkey, Turkmenistan and Uzbekistan (Ouvrard 2016). Moreover, *Psyllopsis machinosus* Loginova, 1963 has also been reported from Afghanistan, Armenia, Caucasus, Georgia, Iran, Kazakhstan, Tadzhikistan, Tunisia, Turkey and Turkmenistan (Ouvrard 2016). In Iran, the first psyllid—*Psyllopsis fraxini* (L. 1758)—has been reported on *Fraxinus oxyphylla* M. Bieb., *Populus euphratica* Olivier, *P. nigra* L., and *P. pyramidalis* L (Burckhardt and Lauterer 1993). The species *P. repens*, *P. securicola* and *P. machinosus* were reported in 1963 (Burckhardt and Lauterer 1993) and the species *Psyllopsis narzykulovi* Bajeva, 1964 was reported in 2014 from Iran (Hesami et al. 2014). The species *P. repens*, *P. securicola*, and *P. fraxini* have been reported from Kerman province (Burckhardt and Lauterer 1993). However, the identification of *P. fraxini* (based on samples collected in 1902) needs to be confirmed (Burckhardt and Lauterer 1993). Also, *P. machinosus* has been reported from Kerman province in 2016 (Lashkari et al. in press).

*Psyllopsis* species have a similar adult morphology (Malenovský and Jerinić-Prodanović 2011), and the adults are usually characterized by male paramere and female proctiger (Burckhardt and Lauterer 1993, Malenovský and Jerinić-Prodanović 2011). Species of *Psyllopsis* also have the same host (genus *Fraxinus* spp.) and biology, as they overwinter in the egg stage and usually have two generations per year (Hodkinson 2009). Moreover, it has been shown that several *Psyllopsis* species can together be encountered on a one-host plant (Loginova 1963).

Morphological differences between males versus females sometimes cause highly intraspecific variation in Psylloidea. These variations have been specially shown in body size and color and also wing size and color (Hodkinson 2009, Brown and Hodkinson 1988, Conci and Tamanini 1983). Sexual dimorphism may arise as size and shape dimorphism or by allometry (i.e., correlation of shape and size) (de Camargo et al. 2015).

Geometric morphometric analysis (GMA) comprises a powerful tool to study size and shape. As a technology, it has been known to detect similarities and differences between morphological structures. Numerous studies have been conducted to describe the geometric differences between different populations in Iran (Khaghaninia et al. 2008, Khiaban et al. 2010, Lashkari et al. 2013, Lashkari and Iranmanesh 2015, Mozaffarian et al. 2005, 2007, Sadeghi et al. 2009, Zahiri 2003). Moreover, several studies have been conducted for the discrimination of some related species (Aghagoli et al. 2013, Gómez et al. 2014, Mitrovski-Bogdanović et al. 2014, Zarei et al. 2013).

In this research, wing shape and size, sexual dimorphism and their allometric effects of the three species of the genus *Psyllopsis* are studied for identification purposes, which are useful in pest management programs. Therefore, the following questions have been set out, to answer whether: 1) wing geometry can help us to identify the three ash psyllids *P. repens*, *P. securicola*, and *P. machinosus*; and, on the other hand, is there a specific wing geometric characteristic for each species? 2) Is there sexual dimorphism in the wing shape and size of the three ash psyllids?

## Materials and methods

### Sampling and preparing the specimens for geometric morphometric analysis

The adults of three species of ash psyllids, *P. repens*, *P. securicola*, and *P. machinosus* were collected from infected ash trees in Kerman province, Iran, in 2015. The place of collection was located in the western part of Kerman province (Bahramjerd) at 29°55'55"N, 56°40'27"E and at 2076 m a.s.l., for *P. repens* and *P. securicola*; and at 29°52'16"N, 56°57'17"E and at 2102 m a.s.l., for *P. machinosus*. The number of specimens for each species was chosen at more than 2P-4, which is equal to the number of variables in partial warp matrix including the uniform component (W matrix), where P is the number of landmarks (Zelditch et al. 2004). Therefore, 60 specimens including adult male and female from each species were randomly selected as sample size, which is more than the number of variables in W matrix. The right forewing of each specimen was used to prepare microscopic slides (using Canada balsam) and the photos were captured by a stereomicroscope, coupled with a digital camera. All the photos were captured with 40× magnification.

### Geometric morphometric analysis

**Figure 1.**
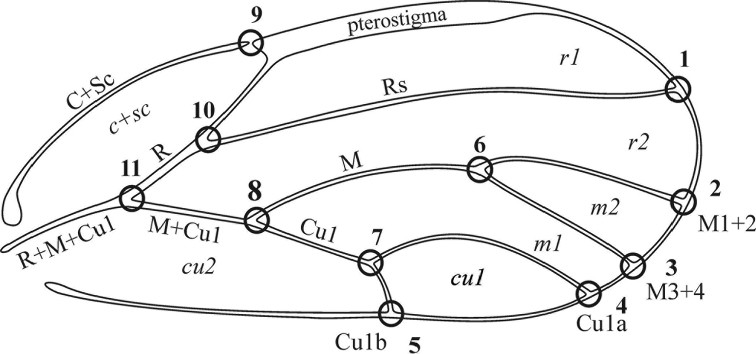
Position of landmarks (circles) in the right forewing of *Psyllopsis machinosus*. Position of landmarks follows that of Lashkari et al. (2013).

In geometric morphometric analysis, a total of eleven homologous landmarks, Type 1, were selected on the forewing (Fig. 1) and digitized by tpsDig program (Rohlf 2015). The aligned individuals were then analyzed using TpsRelw program (Rohlf 2015) and the shape variables, partial warp scores or PWs, which were generated by thin-plate spline equation, were used as a data matrix to compare shapes among the three species. Centroid sizes—as a size measure—were also calculated by tpsRelw (Rohlf 2015) and used to compare the wing size between the three species (Adams and Funk 1997).

One-way MANOVAs were designed to detect any significant differences in wing shape between species. An ANOVA procedure and Tukey pair-wise comparisons were used to detect differences in centroid size between the three species. A regression, shape on size variables, and a MANCOVA were designed to detect any allometric growth and separate allometric trajectories. Statistical analyses were performed in NTSYS-pc (Rohlf 1998) and in SAS statistical program (SAS 2003). Relationships among the three species were described by UPGMA clustering method using NTSYSpc program (Rohlf 1998).

The specimens were deposited in the Psylloidea Collection of the Department of Biodiversity, Institute of Science and High Technology and Environmental Sciences, Graduate University of Advanced Technology, Kerman, Iran.

## Results

### Psyllid sexual dimorphism

In this study, according to the results of MANOVA, significant differences were found in the wing shape of male/female wings in all psyllid species (Tab. 1).

**Figure 2.**
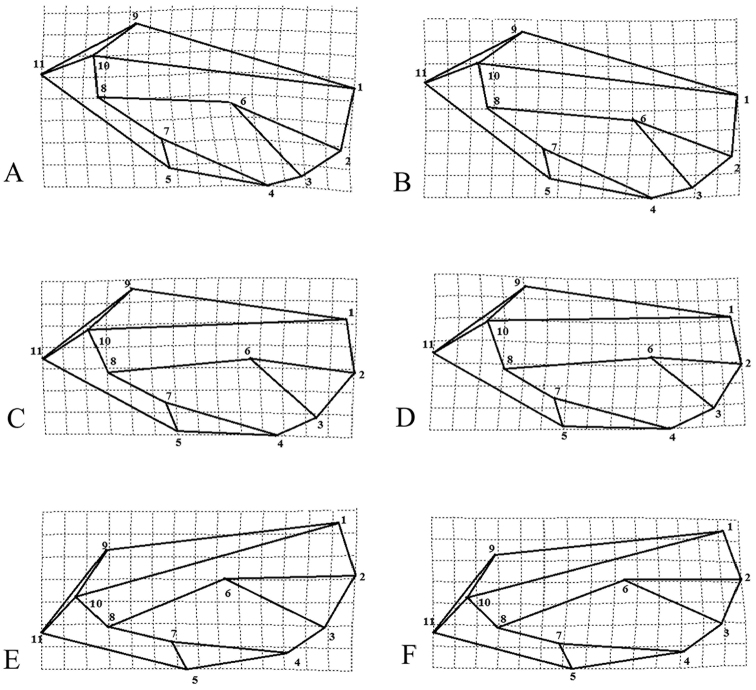
Detected shape differences of forewings in the female and male of *P. machinosus* (**a** Female **b** Male), *P. securicola* (**c** Female **d** Male) and *P. repens* (**e** Female **f** Male).

**Figure 3.**
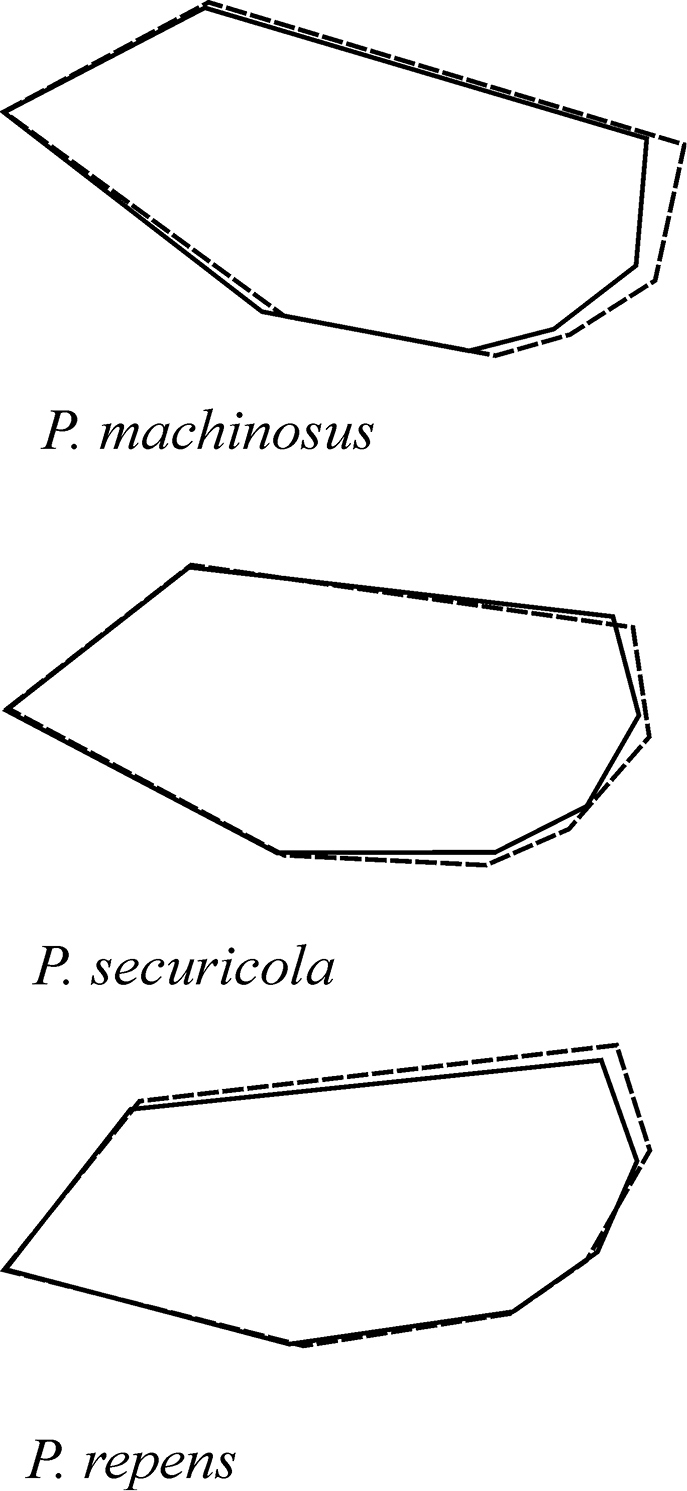
Superimposed forewing shapes of male and females of *P. machinosus*, *P. securicola*, and *P. repens*.

**Figure 4.**
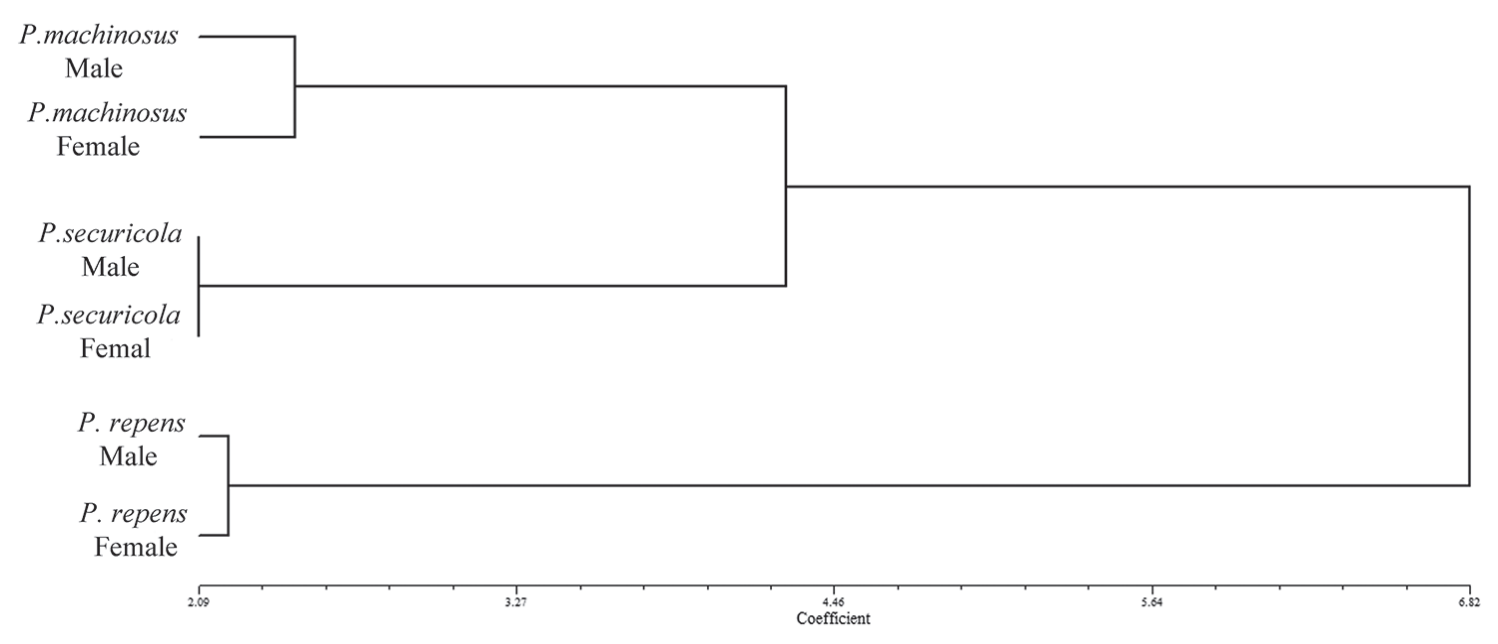
Cluster analysis, using UPGMA method, of the males and females of *P. repens*, *P. securicola*, and *P. machinosus*.

Wings in females of *P. machinosus* and *P. repens* are wider and longer than those in males; the main changes in *P. machinosus* and *P. repens* are related to the veins Rs (Landmark 1) and slightly M1+2 (Landmark 2), which are longer in females and tend to be more longer on apical edges (Figs 2, 3). Though the wing width was not clearly different in males and females of *P. securicola*, it was found that the forewing in females is relatively longer than those in males (Figs 2, 3). The cluster analysis, based on the wing shape variation using UPGMA method, showed that the male and female of each species are placed together. Moreover, *P. repens* was clustered distinctly from *P. securicola* and *P. machinosus* (Fig. 4).

**Figure 5.**
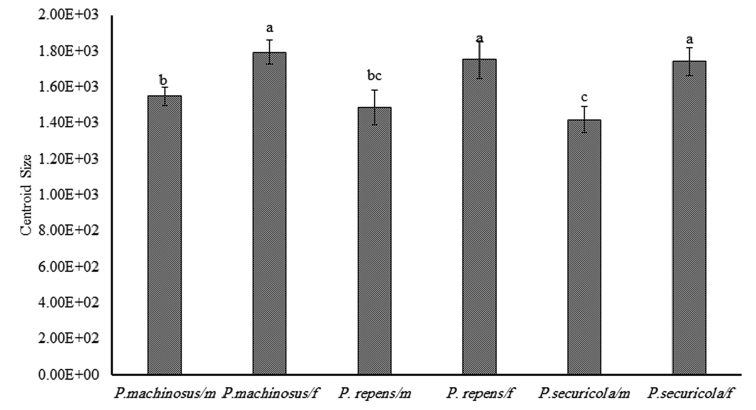
Wing size comparison of the forewing of the males and females of *P. repens*, *P. securicola*, and *P. machinosus*. Means with the same letter are not significant from each other.

The size comparisons showed significant differences between wing size of the female and the male in all the species studied (F = 115.31, P < 0.0001) (Fig. 5).

Allometric analysis showed allometric growth among female and male individuals (Tab. 1A). Though allometric slopes in female and male populations did not vary significantly (Tab. 1B), except *P. securicola*, there were still significant differences between shapes of the wings when size was held constant (Tab. 1C). Therefore, the wing shape of females and males vary in parallel and separate allometric trajectories. It seems that the shape differences observed between the male and the female of *P. securicola* were related to the size.

### Psyllid Identification: Wing shape in *P. repens*, *P. securicola*, and *P. machinosus*

**Figure 6.**
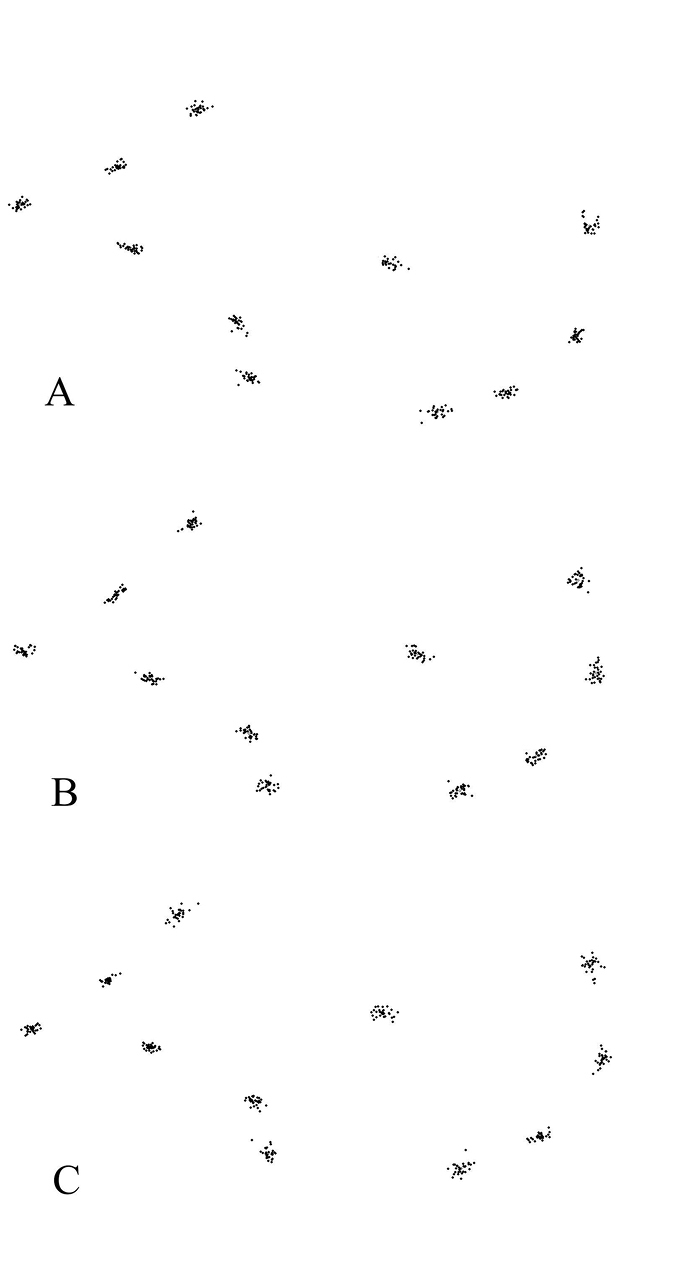
Superimposed landmarks on the forewing of three species of ash psyllid: **A** *P. machinosus* **B** *P. securicola*, and **C** *P. repens*.

The superimposition of the forewing landmarks of the three species of ash psyllid species *P. repens*, *P. securicola*, and *P. machinosus* adults showed a range of variation at each landmark, especially Landmarks 1, 2, and 6 (Fig. 6).

**Figure 7.**
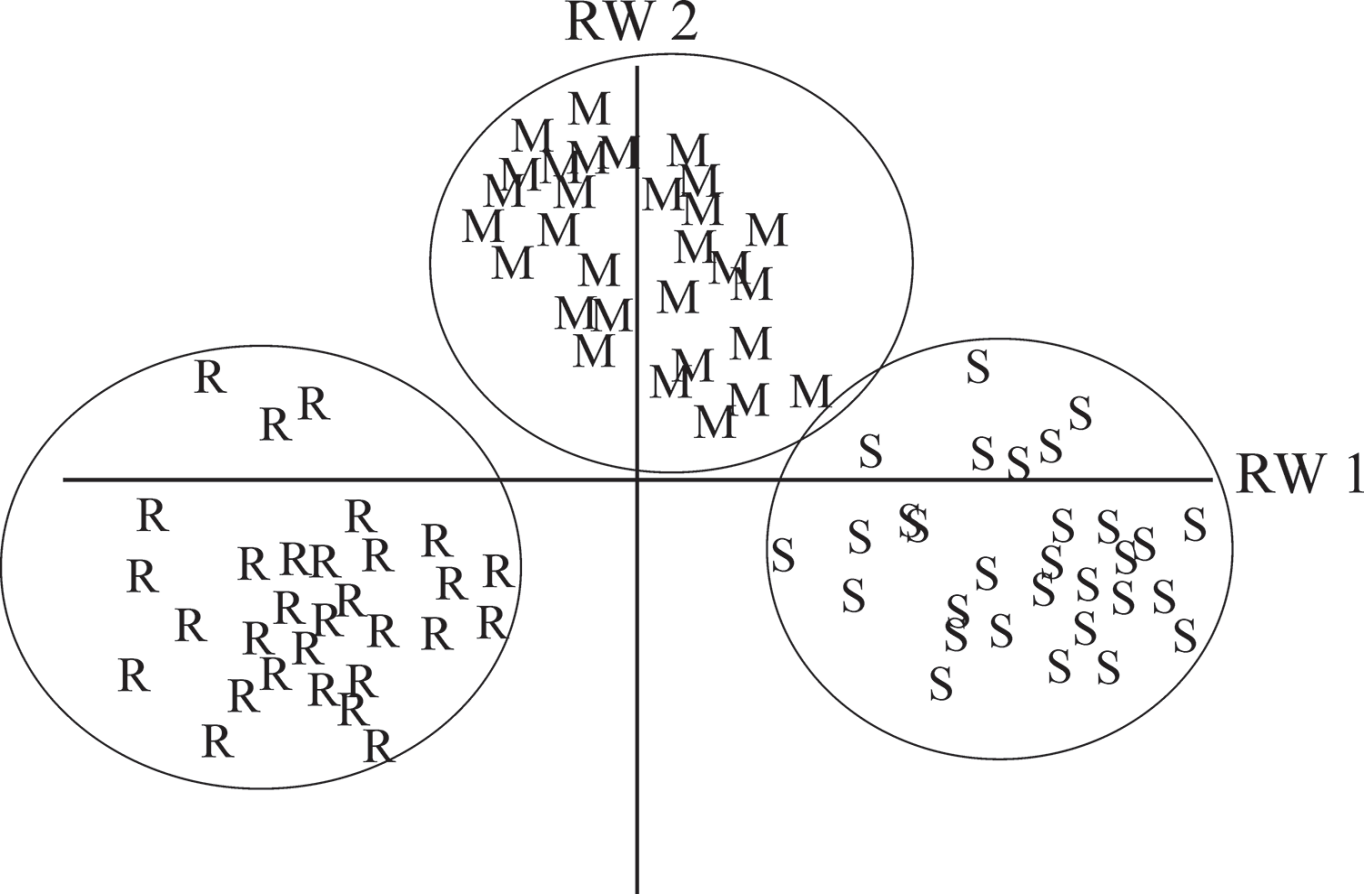
Scatter plot of the first two principal components of the three species of Ash psyllids. Abbreviations: r = *P. repens*, s = *P. securicola*, and m = *P. machinosus*

**Figure 8.**
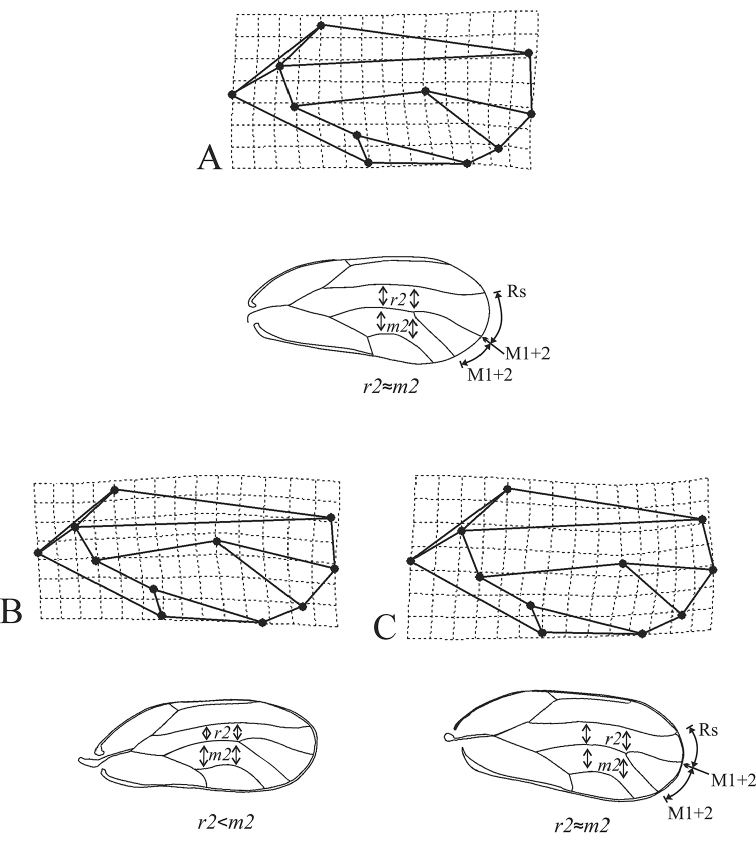
Shape variation along the positive RW2 (**a**), negative RW1 (**b**), and positive RW1 (**c**) extremes for *P. machinosus*, *P. repens*, and *P. securicola*, respectively.

The first two relative warps explained 45.70% and 16.95%, respectively, of the total shape variation between the three species (Fig. 7). The positive and negative extremes of wing shape variation along the first relative warps (RW1) axis are shown in Fig. 8.

As a diagnostic novel character, based on the relative warp visualization plot related to the positive extreme of RW1 (Fig. 8), Landmark 2 (vein M1+2) was closer to Landmark 3 (vein M3+4) in *P. machinosus*, while in *P. securicola*, Landmark 2 (vein M1+2) is located between Rs and M3+4. Moreover, in *P. machinosus* and *P. securicola*, Landmark 6 (junction of M1+2 and M3+4 veins) showed inclination to Landmark 2 (apical part of the wing), which led to increasing distance between Landmarks 6 and 8 (junction of M and Cu1 veins). In other words, the vein M was longer than the M1+2 and also M3+4. In *P. repens*, Landmark 6 was located between Landmark 2 (the apical part of the wing) and Landmark 8; in other words, the vein M, M1+2 and M3+4 were more or less the same in length. Moreover, in *P. repens*, Landmark 6 was closer to Landmarks 1 and 10 (in vertical grids of the relative warp visualization plot) rather than the distance mentioned in *P. machinosus* and *P. securicola*. Therefore, the vein M is closer to the vein Rs rather than Cu1a (above the Landmark 6), or the cell r1 is narrower than the cell m1 above Landmark 6 (junction of M1+2 and M3+4) and defined a concave shape. The vein M, in contrast, is located between Rs and Cu1a in *P. machinosus* and *P. securicola* (above Landmark 6), or the cell r1 and m1 have relatively same width above Landmark 6 (junction of m1+2 and m3+4) and defined a relatively convex shape.

**Figure 9.**
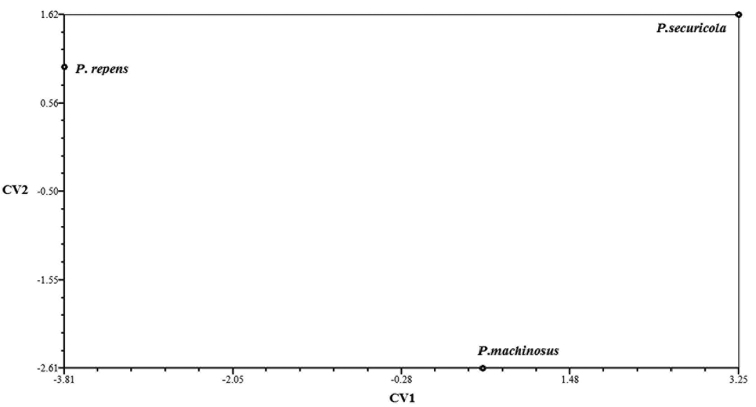
Ordination of the group means along the first two canonical variate axes (CV1 and CV2) based on the generalized distance matrix.

The results of the MANOVA showed that there was a significant difference in the mean wing shapes of the three species of ash psyllids (Tab. 1). Canonical variate analysis confirms these results (Fig. 9). Based on the UPGMA clustering method, *P. repens* was clustered distinctly from *P. securicola* and *P. machinosus*, while *P. securicola* and *P. machinosus* were clustered together (Fig. 4).

### Wing size in *P. repens*, *P. securicola*, and *P. machinosus*

Wing size comparisons between the three species *P. repens*, *P. securicola* and *P. machinosus* showed significant differences (F = 13.74, P < 0.0001). Pairwise comparisons between the three species (using HSD post-hoc test, alpha = 0.01) showed that *P. machinosus* had a larger wing and significant differences with *P. securicola*, while *P. repens* had medium wing size and did not have differences with other species (Fig. 5).

### Allometric analysis in *P. repens*, *P. securicola*, and *P. machinosus*

The results showed allometric growth among the species. Allometric slopes in the three species did not vary significantly, but there were still significant differences between shapes of the wings when size was held constant (Table 1). Therefore, the wing shape of the three species varies in parallel and on separate allometric trajectories.

**Table 1.** MANOVA: Wing shape differences in male versus female within *P. machinosus*, *P. securicola*, and *P. repens* and also between the three species. Allometry test: allometric growth (a), comparing allometric slope (b) and comparing shape in constant size (c) in male versus female within the three species and also between them.

species	MANOVA		Allometry tests					
	Wilks’ Lambda	Prob.	(a) Wilks Lambda	(a) Prob.	(b) Wilks Lambda	(b) Prob.	(c) Wilks Lambda	(c) Prob.
*P. machinosus*	0.2756	<0.0001	0.3914	3.982E-004**	0.6884	0.4996	0.3284	3.348E-005**
*P. securicola*	0.3964	0.0005	0.3753	2.033E-004**	0.7146	0.6191	0.5920	0.1295
*P. repens*	0.4204	0.0012	0.4366	0.0021**	0.5697	0.0982	0.4724	0.0084**
between species	0.0222	<0.0001	0.5598	3.502E-004**	0.6030	0.3785	0.0292	2.180E-037**

** Significant at P < 0.01

### Key to species

**Table:** 

1	The vein M is closer to the vein Rs rather than Cu1a, or the cell r1 is narrower than the cell m1 above the junction of M1+2 and M3+4 (Fig. 4)	***Psyllopsis repens* Loginova**
–	The vein M is located between Rs and Cu1a, or the cell r1 and m1 have relatively the same width above the junction of M1+2 and M3+4 (Fig. 4)	**2**
2	The vein M1+2 is closer to the vein M3+4 (Fig. 4)	***Psyllopsis machinosus* Loginova**
–	The vein M1+2 is located between Rs and M3+4 veins	***Psyllopsis securicola* Loginova**

## Discussion

### Psyllid sexual dimorphism

Many systematic studies of different psyllid groups report larger females/wings and a number of species have been described with qualitatively different male and female wings; for example, the autumn morph of *Pachypsylla japonica* Miyatake, 1968 have darker wing-pattern which shows sexual dimorphism. This sexual pattern dimorphism was also sporadically found in *Euphalerus fossiconis* Brown & Hodkinson, 1988 (Brown and Hodkinson 1988). Moreover, *Crastina loginovae* Conci & Tamanini, 1983 have strong sexual dimorphism in general body coloration (Conci and Tamanini 1983).

In this study, wing shape differences were found when comparing the sexes in the two ash psyllids *P. machinosus* and *P. repens*, even after removing the allometric component. But in *P. securicola*, allometric analysis showed that the observed wing shape variation was due to size effects, i.e., when size effects were constant, there was no difference in the wing shape between the male and the female. Even though the sexual dimorphism in the wing shape has not been ever recorded in the superfamily Psylloidea, it is observed in some insects such as *Chilo suppressalis* (Walker) (Lep.: Pyralidae) (Zahiri, 2003), *Chironomus imicola* Kieffer, 1913 (Dip.: Chironomidae) (McLechlan, 1986), seven moth species of Sphingidae (de Camargo et al. 2015), and *Ectomyelois ceratoniae* (Zeller, 1839) (Lepidoptera: Pyralidae) (Mozaffarian et al. 2007b).

This phenomenon might be affected by nutrition, as Sarafrazi et al. (2004) showed sexual dimorphism in *Eurygaster integriceps* Puton, 1881 (Hem.: Scutelleridae) on wheat, while there was no such difference in the same species on barley (Sarafrazi et al. 2004). Moreover, Mozaffarian et al. (2007b) showed that wing shape is different in the male and the female of *E. ceratoniae* in all selected host plants (pomegranate, fig, pistachio, and walnut).

Different hypotheses have been developed to explain how sexual size allometry can arise. McLechlan (1986) believes that the sexual dimorphism observed in insects is due to different roles of adult males and females, and Dale et al. (2007) categorizes the reasons into three main groups, including: evolutionary constraints, natural selection, and sexual selection. Forewings of females have larger surfaces with rounder apical edge, which are suitable for flight with greater maneuverability (de Camargo et al. 2015). Triangular wingtip (or longer wingtip) in insects can increase energy efficiency during the migratory flight (Lockwood et al. 1998). Bai et al. (2016) showed that populations of grasshoppers with longer wings had better flight capabilities (Bai et al. 2016).

In this study, it was found that forewings in males have smaller surface with straighter apical edges, which suggests friction reduction with air and might result in faster flight and so more efficiency in flights; this might result in finding females. On the other hand, the broader and slower flight in females may result in selecting host plants for oviposition and flying over plants. It is demonstrated that the more elongated wings in butterflies have a positive role in longer spatial movements (Betts and Wootton 1988). In this study, the observed shape and size variations in the females of the species studied may act to optimize flight characteristics and may be able to affect dispersion, migration, search, and selection of host plants. Therefore, more studies on these aspects are required.

The results showed that females of all psyllids studied, *P. machinosus*, *P. securicola*, and *P. repens* have larger wing sizes than those of the males. Sexual dimorphism in size also has not been ever recorded in the ash psyllids. The presence of larger wing size of females may be due to larger body size of the females and affected by the different reproductive role in the males and females (Fairbairn 1997) commonly observed in different insect groups (de Camargo et al. 2015, Fox et al. 2003, Harrison and Cooper 2003; Kraushaar and Blanckenhorn 2002, Mozaffarian et al. 2007, Outomuro and Johansson 2011).

### Psyllid identification

In this study, some areas of the forewings were found to be more variable than others and the prepared key yields correct identification in all studied specimens, which suggests that the wing shape can be useful for identification. The results of GMA suggested that the three species can be discriminated with a high probability of accuracy based on their forewing shapes. Despite the high similarity between the three species, there are some differences in the coordinates of the veins that is useful in their discrimination. The differences are mainly placed in Landmarks 1, 2, 3 and 8, which are related to the veins Rs, M1+2, M3+4, and M, respectively. Based on the results, the obtained characteristics can be used as diagnostic characters to discriminate the three species studied. The other main diagnostic characteristics for the three species are related to the male and female terminalia and also some markings on the thorax, as in *P. repens* they have an anterior lobe and a dorsal incision on the paramere (in profile view) (Burckhardt and Lauterer 1993) and the mesoscutum has four stripes, ranging from light to dark brown (Malenovský and Jerinić-Prodanović 2011), while *P. machinosus* and *P. securicola* have no markings on the head and thorax. *Psyllopsis machinosus* has a forward- and a backward-directed lobe in its male paramere and also female proctiger is subacute apically but *P. securicola* has only the anterior lobe and its female proctiger is truncated apically (Burckhardt and Lauterer 1993).

Little is known about the discrimination of psyllid species and populations using geometric and traditional morphometric methods. In previous studies, the geographic populations of the Asian citrus psyllid (ACP), *Diaphorina citri* Kuwayama, 1908, was investigated by geometric morphometric analyses (Lashkari et al. 2013, Lashkari and Iranmanesh 2015). They showed differences in the wing geometry between ACP populations from Iran, Pakistan, and Florida, and they also found that the apical half of the wing has a more important role in population differentiation than the other part of the wing (Lashkari et al. 2013, Lashkari and Iranmanesh 2015). This is similar to the findings of the current study, which showed that the apical part of the wing (i.e., Landmarks 1, 2, 3) has a more important role in differentiation of species rather the other parts of the wing. The geometric morphometric also has been used to discriminate between three species of grape cicadas (Hem., Cicadidae) in Iran (Aghagoli et al. 2013). They showed that the length ratio of two portions of veins CuA1 and M1+2 were the most important characteristics for distinguishing between the three species, *Cicadatra alhageos* (Kolenati, 1857), *Chloropsalta ochreata* (Melichar, 1902) and *Chloropsalta smaragdula* Haupt, 1920, in the field (Aghagoli et al. 2013). Zarei et al. (2013) studied geometric morphometric of the mandibles in four species of *Calomyscus* Thomas, 1905 (Rodentia: Calomyscidae), including *Calomyscus grandis* Schlitter and Setzer, 1973, *C. elburzensis* Goodwin, 1938, *C. bailwardi* Thomas, 1905 and *C. hotsoni* Thomas, 1920. They demonstrated that the mandible size was not significantly different among the species examined, but the results of geometric morphometric analysis suggested that the four species can be discriminated with a high probability based on their mandibular and first lower molar shapes (Zarei et al. 2013). Alibert et al. (2001) applied quantifying morphological analysis (size and shape) among populations of two forest species of ground beetles, *Carabus auronitens* Fabricius, 1792 and *C. nemoralis* O. F. Müller, 1764, and found a significant shape variation among populations as well as among sexes for both species (Alibert et al. 2001). In another study, species discrimination of some braconid wasps was investigated using geometric morphometrics; their analyses confirmed that the major variation in the wing shape, as described by the first canonical axis, consisted of changes in the length of the radial and m-cu veins, which could be useful characteristics for separation of the species within a ‘dorsale–yomenae’ group (Mitrovski-Bogdanović et al. 2014). Zúniga-Reinoso and Benítez (2015) used geometric morphometric methods to distinguish between two species of tenebrionid beetles (*Nyctelia multicristata* Blanchard, 1846 and *Nyctelia confusa* Zuniga-Reinoso, 2012) that have been traditionally classified as cryptic (Zúniga-Reinoso and Benítez 2015). They showed distinctions between *N. multicristata* and *N. Confuse* (Zúniga-Reinoso and Benítez 2015). A geometric morphometric study was used to analyse intersexual and interspecific variation of size and shape in the mandibles, heads, pronota, and elytra of two sympatric species, *Colophon haughtoni* Barnard, 1931 and *Colophon kawaii* Mizukami, 1996; all measured structures showed significant sexual dimorphism. Males of *C. kawaii* were significantly larger than *C. haughtoni* for all structures. Their results support the species status of *C. kawaii*, which was in doubt due to its hybridization with *C. haughtoni* (Eldred et al. 2016). In another study, Tabatabaei Yazdi and Adriaens (2013) utilized skull shape and size in geometric morphometric analysis to identify four rodent species (*Meriones tristrami* Thomas, 1892, *Meriones persicus* (Blanford, 1875), *Meriones vinogradovi* Heptner, 1931, and *Meriones libycus* Lichtenstein, 1823). The shape and size analyses showed that *M. libycus* can be distinguished because its skull is the largest and the tympanic bulla is relatively the largest, and that *M. tristrami* can be distinguished from the other species base on skull’s morphometric data (Tabatabaei Yazdi and Adriaens 2013).

The above-mentioned characteristics presented in this research can separate the three species studied; the identification of other species is certainly possible especially on the basis of male genitalia. Considering the importance of identification of species and its importance in pest management programs, morphometric studies with all ash psyllid species are suggested.
